# Wide-field retinotopy reveals a new visuotopic cluster in macaque posterior parietal cortex

**DOI:** 10.1007/s00429-020-02134-2

**Published:** 2020-09-02

**Authors:** Samy Rima, Benoit R. Cottereau, Yseut Héjja-Brichard, Yves Trotter, Jean-Baptiste Durand

**Affiliations:** 1 Centre de Recherche Cerveau et Cognition, Université de Toulouse, Toulouse, France; 2 Centre National de la Recherche Scientifique, Toulouse Cedex, France

**Keywords:** Visuotopy, Dorsal, Primates, fMRI, PIP, CIP

## Abstract

We investigated the visuotopic organization of macaque posterior parietal cortex (PPC) by combining functional imaging (fMRI) and wide-field retinotopic mapping in two macaque monkeys. Whole brain blood-oxygen-level-dependent (BOLD) signal was recorded while monkeys maintained central fixation during the presentation of large rotating wedges and expending/contracting annulus of a “shaking” fruit basket, designed to maximize the recruitment of PPC neurons. Results of the surface-based population receptive field (pRF) analysis reveal a new cluster of four visuotopic areas at the confluence of the parieto-occipital and intra-parietal sulci, in a location previously defined histologically and anatomically as the posterior intra-parietal (PIP) region. This PIP cluster groups together two recently described areas (CIP1/2) laterally and two newly identified ones (PIP1/2) medially, whose foveal representations merge in the fundus of the intra-parietal sulcus. The cluster shares borders with other visuotopic areas: V3d posteriorly, V3A/DP laterally, V6/V6A medially and LIP anteriorly. Together, these results show that monkey PPC is endowed with a dense set of visuotopic areas, as its human counterpart. The fact that fMRI and wide-field stimulation allows a functional parsing of monkey PPC offers a new framework for studying functional homologies with human PPC.

**Electronic supplementary material:**

The online version of this article (10.1007/s00429-020-02134-2) contains supplementary material, which is available to authorized users.

## Introduction

In primates, the posterior parietal cortex (PPC) constitutes the end stage of the dorsal visual pathway and as such, it is notably involved in visuospatial and visuomotor functions (Buneo and Andersen 2006; Freedman and Ibos 2018; Hadjidimitrakis et al. 2019a, b). Most of what we know about how those functions are implemented in the PPC emanates from invasive (anatomical and electrophysiological) studies performed in macaque monkeys. They have notably demonstrated the existence of a myriad of structurally and/or functionally distinct areas (Pandya and Seltzer 1982; Colby et al. 1988; Andersen et al. 1990; Lewis and Van Essen 2000), although their precise number and boundaries remain debated (Van Essen 2004). Such invasive studies have led to the view that monkey PPC is only marginally visuotopic, with coarse topographic representations of visual space restricted to the lateral intra-parietal (LIP) area (Blatt et al. 1990; Ben Hamed et al. 2001), a portion of the dorsal prelunate (DP) area (Heider et al. 2005) and the parieto-occipital areas V6 (Galletti et al. 1999a) and V6A (Galletti et al. 1999b). Because these areas appear more as isolated patches than as a structured ensemble, visuotopy has not been considered so far as a useful criterion for parsing monkey PPC.

In striking contrast, several visuotopic areas have been progressively unveiled in human PPC (Sereno et al. 2001; Schluppeck et al. 2005; Silver et al. 2005; Swisher et al. 2007; Konen and Kastner 2008), thanks to the development of functional magnetic resonance imaging (fMRI) techniques for non-invasive retinotopic mapping (Sereno et al. 1995; Engel et al. 1997). These studies have drawn the view of a dense arrangement of abutting visuotopic maps in human PPC (Wandell et al. 2007; Silver and Kastner 2009). Additionally, human homologues of macaque caudal and anterior intraparietal areas (CIP and AIP) have been described, without the description of their visuotopic organization. In their study, Shikata et al. (2008) found human homologs of macaque areas AIP and CIP that were sensitive to surface orientation and located them on the lateral bank of the anterior IPS and on the medial bank of the IPS, respectively. As for the posterior aspect of the IPS (Van Essen and Zeki 1978), little is known either about its visuotopic organisation, or its function. A recent study (Héjja-Brichard et al. 2020) has shown this area to be sensitive to cyclopean stereomotion in macaque monkeys, with a potential human homolog being area POIPS described in (Georgieva et al. 2009).

The apparent discrepancy with results obtained from invasive studies in monkey PPC might partly reflect inter-species differences in PPC functional organization, notably linked to the emergence of specific human skills such as the use of tools (Kaas and Stepniewska 2016; Orban 2016; Kastner et al. 2017). However, it might also betray the advantage of fMRI-based approaches for uncovering the visuotopic organization of higher-order visual areas, where neurons generally exhibit large and coarsely organized receptive fields (RF) (Patel et al. 2010). This second hypothesis has received support in a recent study by Arcaro et al. (2011). By implementing those non-invasive mapping procedures in macaque monkeys, the authors confirmed the visuotopic organization of LIP and DP but, additionally, they revealed two new visuotopic areas, the caudal intra-parietal areas 1 and 2 (CIP1 and CIP2), lying in between V3A and LIP in the latero-caudal portion of the intra-parietal sulcus.

Building on this seminal success and inspired by recent developments in human mapping studies (Pitzalis et al. 2013), we introduce adaptations to the mapping procedure of Arcaro et al. (2011): wide-field visual stimulation with moving and behaviourally-salient objects, to boost the recruitment of PPC neurons. Besides confirming the existence of CIP1 and CIP2, our results show that they form a visuotopic cluster with 2 additional previously unknown areas of the posterior intra-parietal sulcus, PIP1 and PIP2. This PIP cluster is bordered by other visuotopic areas: V3A/DP laterally, V3d/V6/V6A posteriorly and medially and LIP anteriorly. This organization firmly establishes that in macaques, as in humans, the PPC houses a dense arrangement of visuotopic maps.

## Materials and methods

### Animal model

Two adult female rhesus macaques, M01 and M02 (age: 8 and 9 years old, weight = 5.2 and 5.5 kg), were involved in the present study. Animal housing, handling, and all the experimental protocols (surgery, behavioural training and MRI recordings) followed the guidelines of the European Union legislation (2010/63/UE) and of the French Ministry of Agriculture (décret 2013-118). The project was approved by a local ethics committee (CNREEA code: C2EA – 14) and received authorization from the French Ministry of Research (MP/03/34/10/09). The animals were housed together in a large, enriched enclosure and could thus develop social and foraging behaviours. Health inspections were carried out quarterly on these animals. After habituation to the monkey chair and experimental set-ups, animals were surgically implanted with a plastic head-post, sealed to the skull with ceramic screws (Thomas recording) and bone cement (Palacos + Gentamycine, medium viscosity, Heraeus). After a post-surgery period of about 8 weeks, the animals resumed the behavioural training through daily sessions in a passive fixation task. Details of the surgery procedure and behavioural training are provided elsewhere (Supplementary Material in (Cottereau et al. 2017)).

### MRI recordings

Whole-brain images were acquired on a 3 Tesla MR scanner (Phillips Achieva) using a custom 8-channel phased array coil (RapidBiomed) specially designed to fit the skull of macaques while preserving their field of view. High-resolution T1-weighted anatomical images were acquired with an MP-RAGE sequence (repetition time [TR] = 10.3 ms; echo time [TE] = 4.6 ms, flip angle = 8°; voxel size = 0.5 × 0.5 × 0.5 mm; 192 slices). Functional images were acquired with a GE-EPI sequence with interleaved slice acquisition (TR = 2000 ms, TE = 30 ms, flip angle = 90°, SENSE factor = 1.6; voxel size = 1.25 × 1.25 × 1.5 mm, 32 axial slices).

### Experimental set-up and behavioural task

**Fig. 1:**
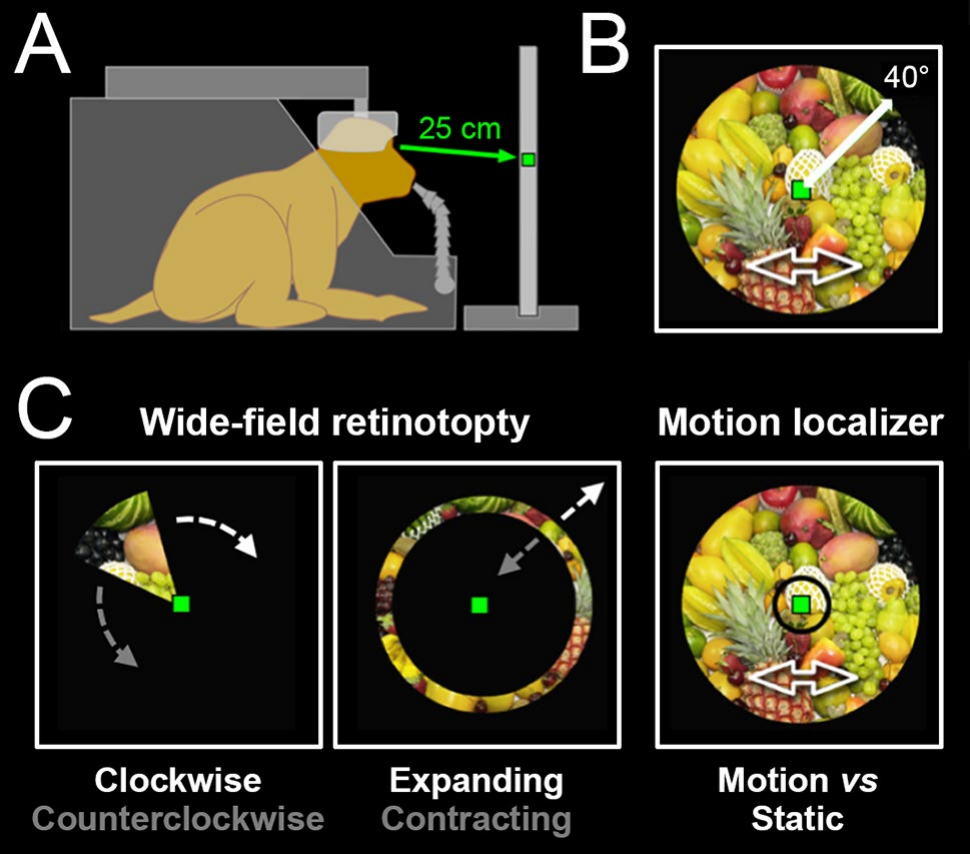
Experimental set-up and protocol. **a** Schematic drawing of a head-restrained macaque in an MRI compatible primate chair, fixating a central green dot located on a screen at a viewing distance of 25 cm. **b** Illustration of the shaking fruits basket video used for wide-field visual stimulation (covering 80° of the visual field). **c** Wide-field retinotopy is done with a wedge aperture rotating either clockwise or counter-clockwise on top of the video for polar angle mapping (left panel) and with an expanding or contracting ring aperture for eccentricity mapping (middle panel). Motion localizer (right panel) was performed with central (< 3°) or peripheral (> 3°) circular aperture on top of either the video (motion condition) or static images extracted from the video (static condition). Monkeys were trained to maintain fixation on the central green dot during visual stimulation

During the scanning sessions, the animals were head-fixed, seated in a sphinx position within their primate chair, into the bore of the magnet (Fig. 1a). They faced a translucent screen at a viewing distance of 25 cm. This short viewing distance allowed the presentation of wide-field stimuli (~ 80° of visual angle), rear-projected on the screen by a video projector (Hitachi, CP_X809). The position of one eye was monitored with an infrared video-based eye-tracker at 60 Hz (ASL). During the acquisition of functional sequences (typically 8 to 12 runs per daily session), the animals were involved in a passive fixation task. They had to maintain their gaze within ± 1.5° of a small green square (0.4° × 0.4°) displayed at the centre of the screen to receive fluid reward. The frequency of reward distribution was progressively increased as long as the fixation was not interrupted, to encourage prolonged fixation periods. Only runs in which animals maintain their gaze on the fixation target for at least 85% of the total run duration were retained for further analyses.

### Visual stimuli

The visual stimuli were displayed on the translucent screen, behind the fixation target and centred on that latter. They consisted in home-made videos (resolution = 700 × 700 pixels, refresh rate = 16 Hz) covering a large portion of the visual field (~ 80° of visual angle) and depicting a fruits basket that seemed to approach or recede in depth (through zooming) while also moving back and forth along both the horizontal and vertical dimensions (Fig. 1b). By using stimuli with (1) a large coverage of the peripheral field of view, (2) coherent motion and (3) objects (fruits) the animals might wish to grasp, we intended to maximize the chances to evoke BOLD activations in the dorsal visual cortex. The EventIDE software (OkazoLab) was used for real-time control of the behavioural task and stimuli presentation.

### Experimental paradigms

Both animals participated in 2 experimental paradigms: (1) retinotopic mapping and (2) motion localizer, both involving wide-field visual stimulation and performed across distinct scanning sessions. These paradigms are now described in more detail.

#### Wide field retinotopic mapping

For the mapping, we used wedges in clockwise or counterclockwise rotation and rings in expansion or contraction. However, instead of being filled with luminance-defined checkerboards, as those generally used to map the early visual cortex, the wedges and rings served as apertures applied on top of our video (Fig. 1c). During the acquisition of a functional sequence, one of the 4 stimulus conditions was shown for a total duration of 230 s (the conditions were interleaved across runs), with the central fixation target always visible. In all cases, the stimuli started with the last 10 s of a cycle, and further accomplished 5 full cycles of 44 s. The wedges had a radius of ~ 40° and an angular extent of 49°, so that every point of the visual field covered by the stimulus was stimulated during 6 s per cycle. The rings had a mean eccentricity varying linearly between 0° and ~ 40°, with a constant width of 11° between their inner and outer borders (leading again to 6 s of stimulation per cycle for each point covered by the stimuli). For monkey M01 (M02), we have kept for further analyses 24/25 (24/26) runs for clockwise/counterclockwise wedges and 23/24 (24/26) runs for expanding/contracting rings, collected over 7 (16) distinct sessions.

Motion localizer. The motion localizer consisted in four visual conditions: central (< 3° of retinal eccentricity) and peripheral (> 3°) portions of the fruits basket video shown with intact motion or with static images sampled randomly from the video and refreshed at 1 Hz to minimize visual adaptation (Fig. 1c, right-hand panel). All 4 conditions (Central–Motion, Central-Static, Peripheral-Motion and Peripheral-Static) lasted for 6 s and were surrounded by 10 s periods of blank screen (Baseline condition). The four visual conditions were repeated three times during a run, in a presentation order that varied between the runs. In total, each run lasted 202 s, during which the central fixation target was always visible. Motion sensitivity in the central and peripheral fields of view was assessed by contrasting the visual conditions (Central-Motion > Central-Static) and (Peripheral-Motion > Peripheral-Static), across voxels with significant visual activations ([Central-Motion + Central-Static + Peripheral-Motion + Peripheral-Static] > 4 × Blank Baseline). In total, 18 (25) runs of motion localizer collected over 3 (5) sessions were kept for further analyses in monkey M01 (M02).

### Data processing

#### *Retinotopic mapping: volume*-*based preprocessing of the functional data*

During a preliminary session, functional (GE-EPI, *n *= 300) and anatomical (T1, *n *= 4) volumes were collected for each animal under slight anaesthesia (see Cottereau et al. 2017 for details about that procedure). Functional volumes were averaged into an individual functional template and anatomical volumes were averaged into an individual anatomical template. Affine and non-rigid normalization parameters bringing the functional template onto the anatomical template were estimated from the grey matter maps of both templates, using the normalization tools of the SPM12 software. During the following sessions, only functional volumes were acquired for being pre-processed run by run. They were first slice-time corrected to compensate for the delay caused by the sequential (interleaved) acquisition of the slices. A mean image was then generated for each run for co-registration with the individual functional template. Co-registration parameters were then combined with the normalization parameters transforming the individual functional template to the individual anatomical template. Those combined parameters were then applied to all the functional images of the run in a single interpolation step, which was also used to resample the functional volumes to 1 × 1 × 1 mm voxels. No smoothing was applied to the volumetric data. Rigid realignment between the functional volumes was also omitted since the animal’s head was immobilized by the head-post. Non-rigid deformations could arise, principally caused by sudden postural changes of the animal within its chair. To regress out the signal fluctuations caused by such events, time courses of voxels outside the brain (muscles, eyes, etc.) were extracted and the 10% showing the highest temporal variance were submitted to principal component analysis (PCA) after z-score normalization. The 18 first PCA components were used to regress out all signal fluctuations correlating with those noise regressors within the brain voxels. The number of PCA components was determined empirically, as the one leading to the highest number of cortical surface nodes for which the pRF could be mapped across the 2 animals (see below).

#### *Retinotopic mapping: surface*-*based processing of the functional data*

Models of the left and right cortical surfaces were generated for each individual with the Caret software (Van Essen et al. 2001) based on the grey/white matters segmentation of the high-resolution anatomical images (T1). Functional data were then projected from volume space to surface space as follows. For all surface nodes, 7 sampling points were computed along the normal vectors (from − 0.75 mm to + 0.75 mm), to account for cortical thickness (1.5 to 2.5 mm in macaques). For each node and each run, time courses for the 7 sampling points were extracted by trilinear interpolation from the functional volumes. They were first converted to percent signal change and then averaged in a single mean time course attributed to the surface’s node. Finally, all the time courses belonging to a same node and same type of run were averaged (Supplementary Fig. 1a).

#### Retinotopic mapping: population receptive fields (pRFs) analysis

The BOLD time courses evoked by periodic ring and wedge stimuli are generally processed with a phase-encoding method that is performed in the frequency domain after a Fourier transform (Sereno et al. 1995; Engel et al. 1997). However, a growing number of research groups now privilege approaches based on pRFs analysis (Dumoulin and Wandell 2008; Kay et al. 2013) because they provide better estimations of retinotopic properties than conventional approaches (Dumoulin and Wandell 2008; Kay et al. 2013; Alvarez et al. 2015). The greater robustness of this approach, combined with the fact that it provides additional information about the size of the pRFs, leads us to favor this newer type of analysis. Because our stimuli were frequency encoded, we used the fact that responses to this type of stimuli always have a spectral content constrained to the fundamental frequency (f0 = 1/44 Hz) and its harmonics (nf0 where n is an integer) to filter the data at those frequencies before the pRF analysis. This filtering was performed in the frequency domain. The PRF analysis was then conducted with the publicly available analyzePRF toolbox for matlab (Kay et al. 2013). Following Dumoulin and Wandell (2008), the PRFs were modelled as simple isotropic Gaussian envelopes. Basically, the hemodynamic response functions that have been estimated for both M01 and M02 (Supplementary Material in (Cottereau et al. 2017) were convolved with regressors derived from a large combination of PRFs positions (40401 positions covering the central 80° of the visual field) and sizes (55 sigma values ranging from 0.25° to 35°). Such operation produces a huge repertory of more than 2.2 million theoretical time courses. For each node, the PRF parameters attached to the theoretical time course showing the highest Pearson correlation coefficient (r) with the actual time course was retained. Threshold was set at *r* > 0.5, which corresponds to a t value = 5.35 and an uncorrected one-tailed p value < 10–6 (assuming that for each of the 4 types of run, the filtering collapses the 5 cycles, ending up with a correlation involving 88 samples: 1 cycle = 22 TR × 4 types of run). The signal filtering and modeling procedures are illustrated in Supplementary Fig. 1b and the final r-score maps are shown in Supplementary Fig. 2. Additionally, we assessed the robustness of the model parameters by performing the same analyses on the odd and even runs separately and by comparing the obtained results. As shown in Supplementary Fig. 3, excellent reproducibility was observed, confirming the robustness of our approach. The percentages of PRFs in contralateral space or very close to it (polar angle < 5° or eccentricity < 2°) were 97.8% (96.4%) and 99.0% (97.4%) for the left and right hemispheres of M01 (M02). Only those PRFs were retained to build our polar angle, eccentricity and size maps.

#### Retinotopic mapping: group results on the F99 monkey template

The left and right cortical surfaces of M01 and M02 were morphed on the right cortical surface of the F99 monkey template using a non-rigid iterative closest point algorithm (reversal was first applied along the ‘x’ dimension for the individuals’ left cortical surfaces). Then, for each type of PRF map (polar angle, eccentricity and size), we projected the 8 samples obtained from the odd/even runs in the left/right hemispheres of the M01/M02 monkeys on that template. These samples were finally averaged to obtain group results on the F99 template.

### Motion localizer

Functional images were pre-processed run by run in a way similar to that described for retinotopic mapping. Additionally, functional images were slightly smoothed spatially with a Gaussian kernel (full width at half maximum of 2 mm). Statistical analyses were performed within the framework of the General Linear Model (GLM) as implemented in SPM12, with the 4 visual conditions and the baseline condition as principal regressors and 18 noise regressors derived from a PCA analysis similar to that described for retinotopic mapping.

## Results

We have investigated the visuotopic organization of primate PPC with fMRI in 2 behaving macaque monkeys. Visuotopic mapping is classically performed with checkerboard patterns seen through wedge or ring apertures of moderate size (10-20° of retinal eccentricity). The PPC is a key player of the dorsal where/when pathway. As such, it is known to track the motion, location and saliency of objects, with emphasis on the periphery of the visual field. To maximize the probability of activating the PPC, we have replaced classical retinotopic mapping stimuli with a wide-field “shaking fruit basket” video, seen through much wider ring and wedge apertures (up to 40° of retinal eccentricity; see Fig. 1). Population receptive fields (PRFs) analysis (Dumoulin and Wandell 2008; Kay et al. 2013) was applied to data filtered in the frequency domain (see Materials and methods) to increase signal-to-noise ratio (see Materials and methods). Analysis were performed on spatially unsmoothed data after projection on cortical surface reconstructions.

### Early dorsal visual areas

**Fig. 2:**
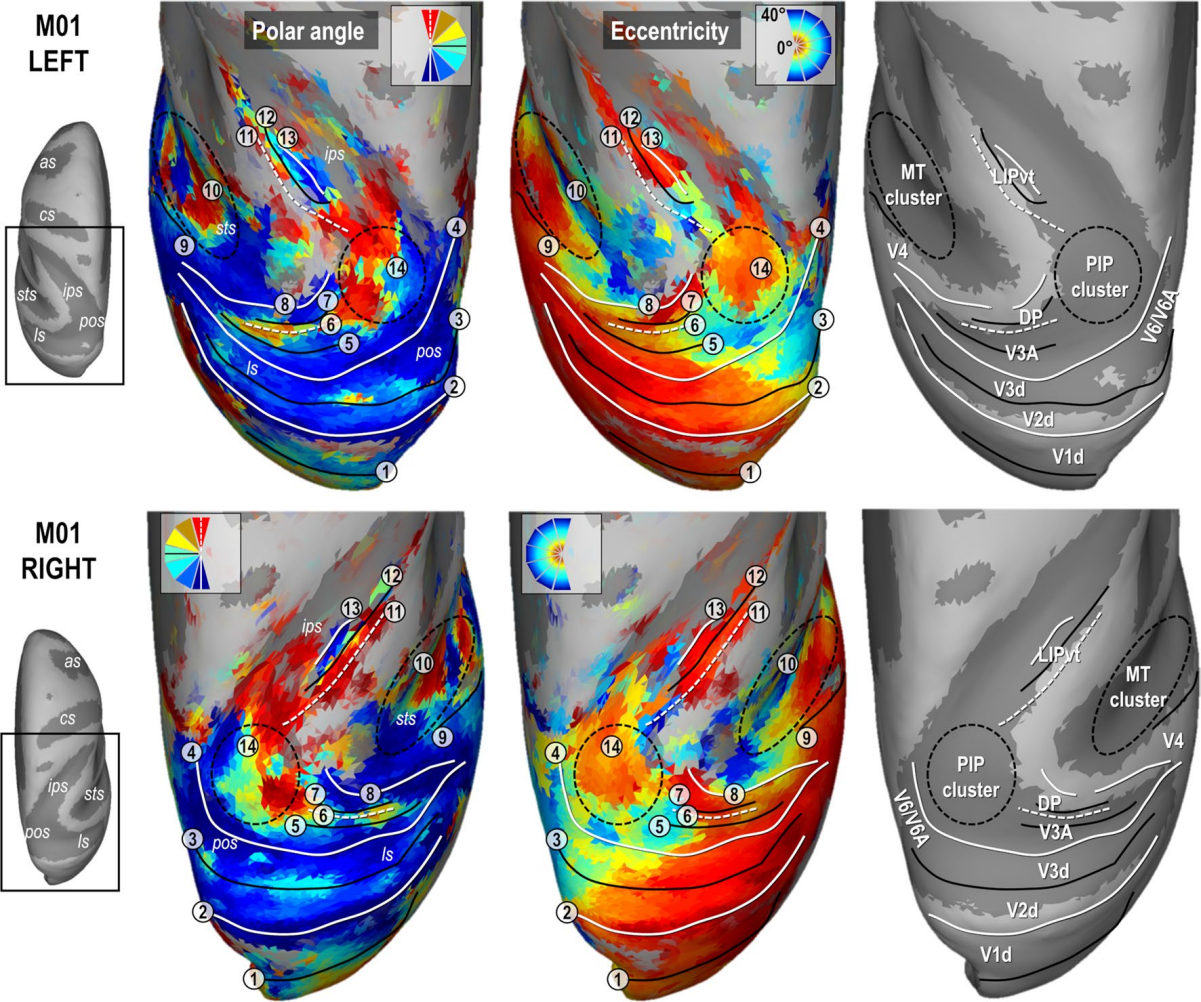
Polar angle, eccentricity maps and cortical frontier projections in monkey M01. Thresholded PRF results (*r* > 0.5; see Materials and methods) are shown on inflated surface reconstructions of the dorsal visual cortex in the left (top panel) and right (bottom panel) hemispheres (LH and RH) of M01. Polar angle maps are on the left panel. The colour code reflects the proximity of the PRFs with the upper-vertical (blue), horizontal (green) and lower-vertical (red) meridians of the visual field. Solid white lines, solid black lines and dotted white lines, further signal those meridians respectively, for delineating the various visual areas. Eccentricity maps are in the middle panel. The colour code progresses from the foveal (red), through parafoveal (yellow), intermediate (turquoise) and eccentric (dark blue) location of the PRFs with respect to the visual field centre. Together with the configuration of meridians, reversal in eccentricity gradients have been used to delineate the MT cluster, anterior to area V4 and the newly defined PIP cluster (black dotted ellipses). Each number corresponds to a frontier. The right most panel represents those frontiers projected on an inflated reconstruction indicating sulci (dark grey) and gyri (bright grey) of the dorsal visual cortex)

**Fig. 3:**
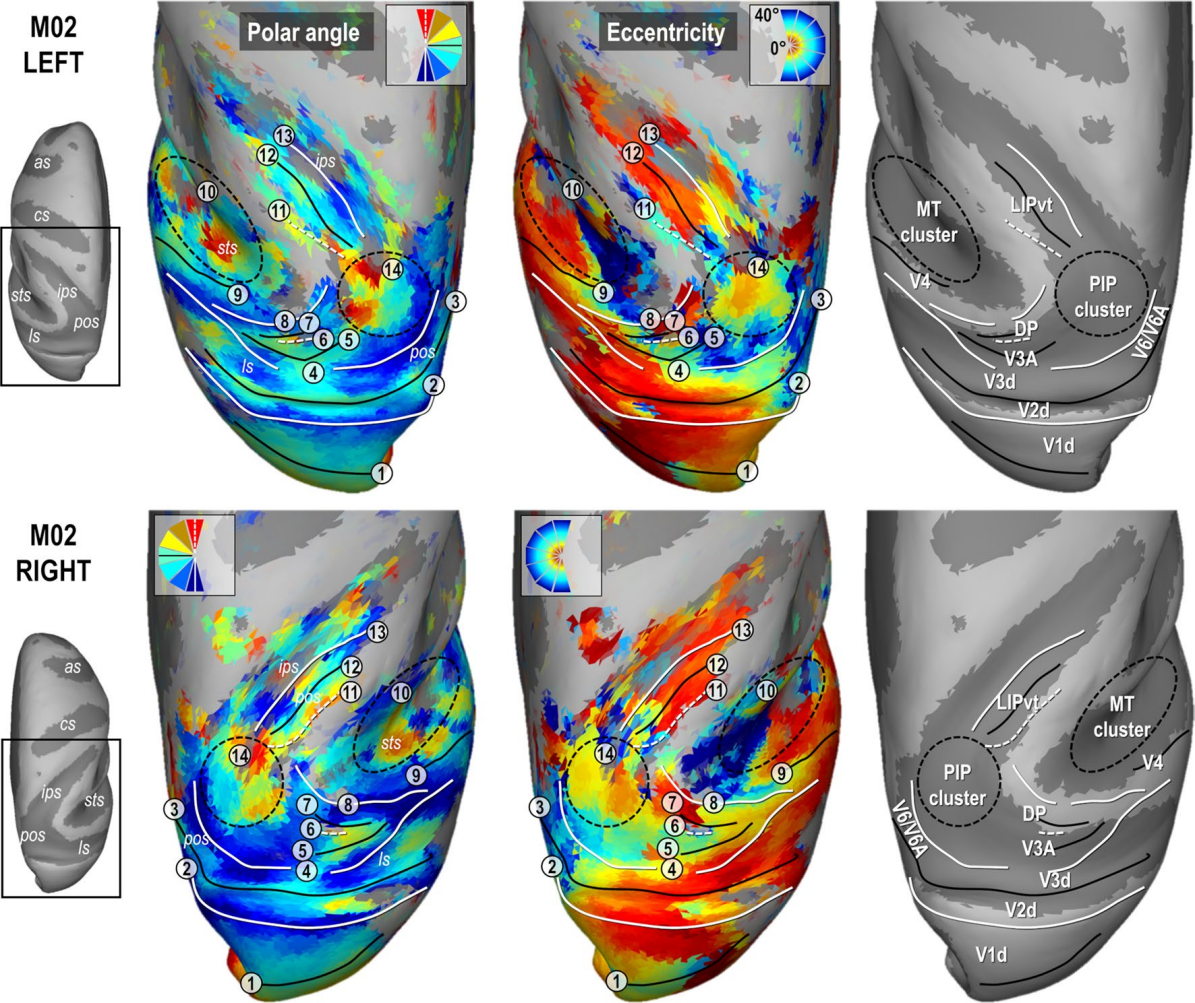
Polar angle, eccentricity maps and cortical frontier projections in monkey M02. Same conventions as Fig. 2

Figures 2 and 3 present the polar angle and eccentricity maps of the PRFs on inflated reconstructions of the dorsal visual cortex in monkeys M01 and M02 respectively. PRFs sizes for both animals are provided in Supplementary Fig. 4. Starting from the occipital lobe in both animals, a representation of the horizontal meridian of the visual field (HM, green color code and solid black line in the top in the polar angle maps of Figs. 2 and 3) distinguishes the dorsal aspect of the primary visual area (V1d) from its ventral aspect (meridian n°1 in Figs. 2 and 3). A representation of the lower vertical meridian (LVM; red colour code and dashed white line) defines the frontier between the dorsal aspects of the primary and secondary visual areas (V1d/V2d; meridian n°2), while the V2d/V3d frontier is identified by a representation of the HM (meridian n°3). Laterally, V3d and V4 are separated by a LVM (meridian n°4). Anterior to V4 in the superior temporal sulcus, the MT cluster recently described by Kolster et al. (2009, 2014) is clearly observable (cluster n°10). It encompasses 4 visuotopic areas (MT, V4t, MSTv and FST) with a foveal confluence (red colour code in eccentricity maps of Figs. 2 and 3) but with nonetheless distinct representations of both lower (green to red) and upper (green to blue) quadrants of the contralateral visual hemi-field.

### Parieto-occipital complex V3A/DP

Anterior to V3d, V3A and the Dorsal Prelunate (DP) area appear to form a cluster with a lateral foveal confluence and 2 mirror representations of the contralateral hemi-field. This organization entails a postero-anterior and latero-medial gradient of polar angle from upper to lower quadrant (blue to green to red; meridians n°4, 5 and 6) in V3A, and from lower to upper quadrant (blue to green to red; meridians n° 6, 7 and 8) in DP, although the DP gradient is mostly observable in both hemispheres of M01. The exact lateral demarcation of the V3A/DP complex is difficult to operate in both monkeys, partly because DP lacks a clear eccentricity gradient. Altogether, these observations fit remarkably well with those of Arcaro et al. (2011), as well as with earlier electrophysiological investigations (Van Essen and Zeki 1978). We note that an alternative interpretation of V3A has been proposed recently (Zhu and Vanduffel 2019), where this area (called R3) is described as being adjacent to V2d, instead of V3d.

### Parieto-occipital complex V6/V6A

**Fig. 4:**
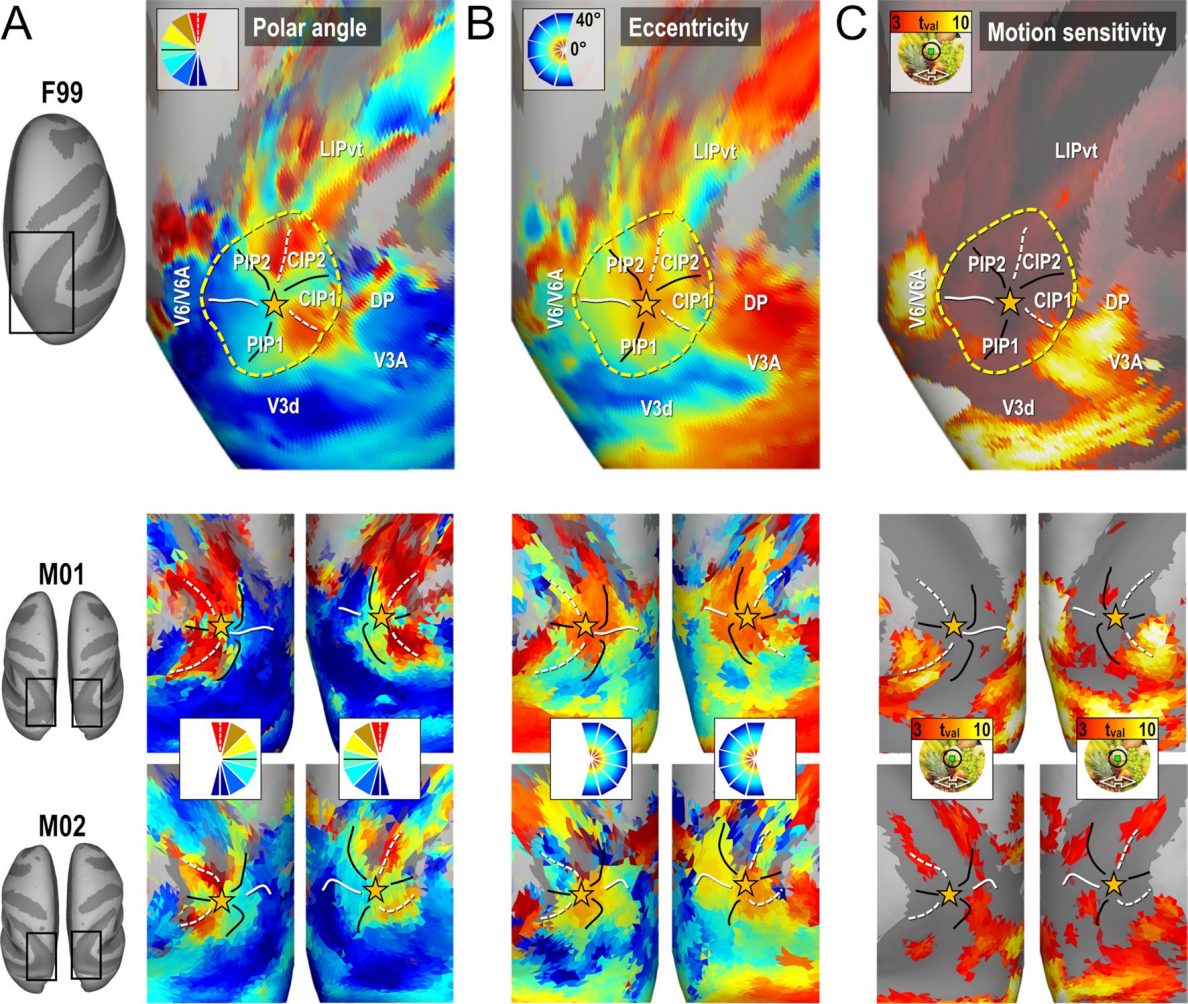
Functional delineation of the PIP cluster. Group (upper line) and individual (lower line) maps of polar angle (**a**), eccentricity (**b**) and motion sensitivity (**c**) in and around the PIP cluster. The cluster is defined by its external borders (yellow dotted ellipses), internal frontiers (meridian representations) and foveal confluence (yellow stars)

Medial to V3d, the anatomical and electrophysiological investigations of Gamberini et al. (2015 for review) have evidenced two areas, V6 and V6A, occupying medial locations along the floor and anterior wall of the parieto-occipital sulcus. Some characteristics of the V6/V6A complex are clearly observable in our data. For instance, in all four hemispheres, the anterior wall of the parieto-occipital sulcus shows an eccentricity gradient ridge, together with a representation of the vertical meridian (principally its lower component), which have been described as marking the frontier between V6 and V6A. In addition, the dorso-anterior aspect of V6A has been shown to house a representation of the central-to-intermediate visual field, which is also consistently found in our data. Finally, the V6/V6A complex has been implied in the processing of visual motion, and our motion localizer clearly indicates the strong motion sensitivity of this cortical sector, as visible in Fig. 4c. Medially, no clear demarcation between V3d and V6 could be delineated, confirming a recent report (Hadjidimitrakis et al. 2019a, b) of a continuous V3d/V6 eccentricity gradient. According to an older electrophysiological description of this boundary (Galletti et al. 1999a, b), no reversal in polar angle or eccentricity gradients is to be expected, compromising the ability to robustly identify this boundary based on polar angle or eccentricity maps alone. While our motion localizer couldn’t provide clear-cut evidence regarding the exact location of this boundary, a gradient reversal of PRF sizes could indicate a potential frontier between V3d and V6. Nevertheless, this point deserves further investigation. In the present study, we thus indicate the location of this complex but with no attempt to clearly define its borders. Only the V6A component could be more safely delineated in all 4 hemispheres. Its MNI coordinates and surface coverage are provided in Table 1.

**Table 1:** MNI coordinates (x, y, z in mm) and cortical surface coverage (mm2) for the PPC visuotopic areas in the left and right hemispheres of M01 and M02

		Left hemisphere				Right hemisphere			
		X	Y	Z	mm^2^	X	Y	Z	mm^2^
PIP1	M01	− 7	− 5	25	42	7	− 5	25	42
	M02	− 9	− 9	24	63	8	− 8	25	47
PIP2	M01	− 5	− 3	28	62	6	− 3	28	76
	M02	− 6	− 9	27	78	6	− 8	28	59
CIP1	M01	− 7	− 6	30	11	8	− 5	29	9
	M02	− 8	− 9	29	14	8	− 8	29	8
CIP2	M01	− 8	− 3	30	18	7	− 3	29	12
	M02	− 7	− 7	29	9	7	− 6	30	13
LIP	M01	− 14	1	38	62	14	3	31	71
	M02	− 11	− 3	32	54	12	− 2	33	67
V6A	M01	− 3	− 9	31	56	4	− 9	31	43
	M02	− 4	− 11	33	46	4	− 11	33	36

### Parietal area LIP

In all 4 hemispheres, a visuotopic organization was observed along most of the lateral bank of the intra-parietal sulcus, which is known to house the lateral intra-parietal (LIP) area. Consistently, this organization reveals an antero-posterior and slightly latero-medial gradient of eccentricity, with the antero-lateral and postero-medial sectors holding respectively representations of the central and peripheral visual field. Polar angle maps indicate that the lower and upper visual field quadrants are located antero-medially and postero-laterally, respectively (meridians n° 11, 12 and 13). Overall, this visuotopic portion of LIP (LIPvt) fits quite well with those described in previous monkey fMRI studies (Patel et al. 2010; Arcaro et al. 2011). Its MNI coordinates and surface coverage are provided in Table 1. Posterior to LIPvt, and anterior to V3d/V3A, our polar angle and eccentricity maps reveal a complex but consistent visuotopic organization which is now described in more details (i.e. the posterior intra-parietal or PIP cluster, n°14 in Figs. 2 and 3).

#### Parietal cluster PIP

The upper line of Fig. 4 shows the average maps of polar angle and eccentricity across the four hemispheres of M01 and M02, on top of the right cortical surface of the template monkey F99. These maps reveal that in between V3d/V3A posteriorly and LIPvt anteriorly, there is a circular succession of visual field meridian representations which merged into a shared representation of the central visual field, indicated by a star in Fig. 4. Altogether, this organization seems to form two distinct representations of the contralateral lower quadrant of the visual field laterally (CIP1 and CIP2) and 2 full representations of the contralateral hemi-field medially (PIP1 and PIP2). Importantly, this particular organization is also observed at the level of each individual maps, as shown in the lower line of Fig. 4. Interestingly, Fig. 4 also reveal that the medial and lateral borders of this PIP cluster, with V6/V6A and V3A/DP respectively, are marked not only by an inversion of the eccentricity gradient, but also by the much stronger motion sensitivity of these bordering areas.

#### Parietal cluster PIP: CIP1 & CIP2

**Fig. 5:**
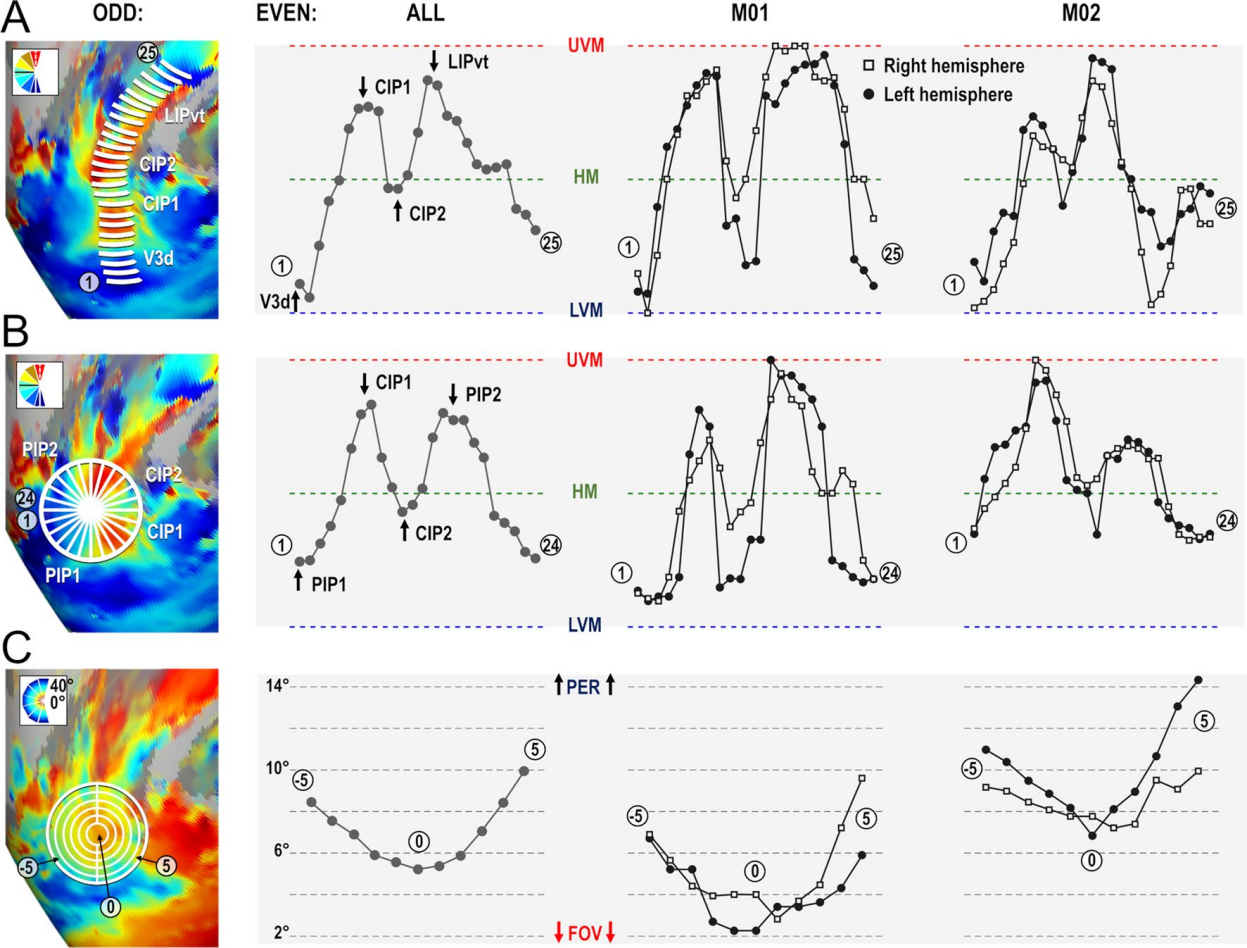
Polar angle and eccentricity profiles of areas of the PIP cluster. **a** Polar angle profile from V3d to LIPvt. The leftmost panel shows line segments drawn on top of the 4 cortical surfaces processed from odd runs, forming paths going from V3d posteriorly to LIPvt anteriorly. Using the coordinates of these segments, we plotted the polar angle profiles from the even runs. These profiles were highly consistent across animals (2 rightmost panels) and hemispheres (square and round markers), with gradient inversions marking the frontiers between V3A/CIP1, CIP1/CIP2 and CIP2/LIPvt. **b** Polar angle profile of the PIP cluster. Again, we used polar maps from odd runs to divide the PIP cluster into 24 spokes. We then used the location of the spokes drawn on the odd run polar angle results to draw the profile on even run polar angle maps. Gradient inversions mark the frontiers between CIP2/CIP1, CIP1/PIP1, PIP1/PIP2 and PIP2/CIP2 when progressing from anterior-to-lateral-to-posterior-to-medial sectors. These results were also consistent across animals and hemispheres. **c** Eccentricity profile of the PIP cluster. We used eccentricity maps from odd runs to draw 5 concentric rings with the foveal representation of the PIP cluster as the origin. We then used the coordinates of rings to extract the eccentricity profiles from even runs. The profiles show a clear evolution of visual field eccentricity from the foveal representation in the fundus of sulcus, extending outward to the surrounding gyri. These results were also consistent across animals and hemispheres

A major finding of Arcaro et al. (2011) concerned the existence of two new visuotopic areas in the caudal portion of the intra-parietal sulcus, CIP1 and CIP2. By studying the evolution of polar angle gradients between V3A posteriorly and LIP anteriorly, the authors identified a succession of gradient reversals signaling transitions between V3A/CIP1, CIP1/CIP2, and CIP2/LIP. To compare this observation to our present results, we followed a similar approach by drawing small segments parallel to the polar angle gradient and perpendicular to the eccentricity gradients between V3A and LIPvt using the averaged polar angle and eccentricity maps computed from the even runs of all four hemispheres (white line segments in the left most panel of Fig. 5a). Then, along each segment, we computed the average polar angle value from the odd runs (i.e. independent data set) to construct the profiles shown in Fig. 5a. In addition to showing high consistency between the four hemispheres and even-to-odd run transferability, these profiles are also strikingly similar to those shown in Fig. 4 of Arcaro et al. (2011). The limits between V3A and CIP1 and between CIP2 and LIPvt are marked by representations of the UVM, while the limit between CIP1 and CIP2 is defined by a representation of the HM. Thus, these latter areas mostly represent the upper quadrant of the contra-lateral hemifield, as already pointed out by Arcacro et al. (2011). Altogether, these polar angle analyses bring firm confirmation for the existence of visuotopic areas CIP1 and CIP2. However, according to Arcaro et al., these areas share a foveal representation lying on the lateral side. Rather, our results show a consistent representation of the central visual field along their medial side, close to the fundus of the intra-parietal sulcus. This point is very important, since it starts drawing a more extended cluster definition, in which this foveal representation is shared not only by CIP1 and CIP2 laterally, but also by 2 additional and newly defined posterior intra-parietal areas, PIP1 and PIP2, extending medially. Further evidence for this cluster organization is provided in the following section.

#### Parietal cluster PIP: PIP1 & PIP2

Average maps drawn from half of the runs were also used to draw a circular area encompassing CIP1/2 laterally and centered on their medially-located central visual field representation, as illustrated in the left-most panel of Fig. 5b. Circular polar angle profiles were then constructed by subdividing the circular area into 24 equal sectors and by averaging the polar angle values of all the nodes encompassed within a sector, yielding 24 mean polar angle values computed from the other half of the runs. The three right-most panels of Fig. 5b present those profiles for the group and for each of the four hemispheres. They clearly establish that besides the gradient inversions defining the mirror lower quadrant representations of CIP1 and CIP2 laterally, other inversions reveal 2 mirror representations of the full contra-lateral hemi-field medially. We have named those newly identified visuotopic maps posterior intra-parietal areas 1 and 2 (PIP1/2). Both CIP1/PIP1 and CIP2/PIP2 share a UVM representation, while PIP1/PIP2 share an LVM representation. Thus, both PIP1 and PIP2 hold complete representations of the contralateral hemi-field. Importantly, these circular profiles of polar angle were very similar when considering separately the central, intermediate and more distant portions of the 24 sectors, indicating that the polar angle gradients are roughly perpendicular to the spokes of the sectors (see Supplementary Fig. 5). To further assert the cluster organization of these areas, surface nodes belonging to the lateral and medial aspects of the same circular area were segregated before being subdivided in five eccentric rings with the cluster’s foveal confluence as the origin. Mean PRF eccentricity was computed for each of these 10 half circles (5 for medial, 5 for lateral). The rightmost panels of Fig. 5c shows clear gradients of increasing eccentricity as distance of the surfaces’ nodes from the foveal confluence increases, both laterally and medially. The nearly three times wider range of retinal eccentricities covered by our mapping stimuli (40° against 15° in that previous study) may account for the elucidation of this centripetal eccentricity gradient). Importantly, the data used to draw the lines, wedge segments, and eccentric circles to assess the profiles of the different areas of the PIP cluster was independent from the data we tested. Indeed, the coordinates were extracted from the odd fMRI runs, while the polar angle and eccentricity values were extracted from even fMRI runs.

### Parietal cluster PIP: overall anatomo-functional definition

Enlarged views of this newly identified visuotopic cluster are shown on top of the maps of polar angle (Fig. 5a), eccentricity (Fig. 5b) and motion localizer (Fig. 5c) with the star indicating foveal confluence. In most hemispheres, the external borders of the PIP cluster are defined both by representations of visual field meridians and eccentricity gradient inversions. Actually, it shares UVM representations with both V3A/DP laterally and LIPvt anteriorly, and a LVM representation with V3d/V6/V6A posteriorly and medially. It also shares peripheral field representations with these neighboring occipital and parietal areas. Additionally, the postero-medial border between the PIP cluster and the V6/V6A complex is marked functionally by a strong motion sensitivity of V6/V6A, contrasting with the lack of motion sensitivity in PIP1/2. The stereotaxic coordinates and cortical surface coverage of these different visuotopic areas are provided in Table 1.

### Visual field coverage of PIP cluster areas

**Fig. 6:**
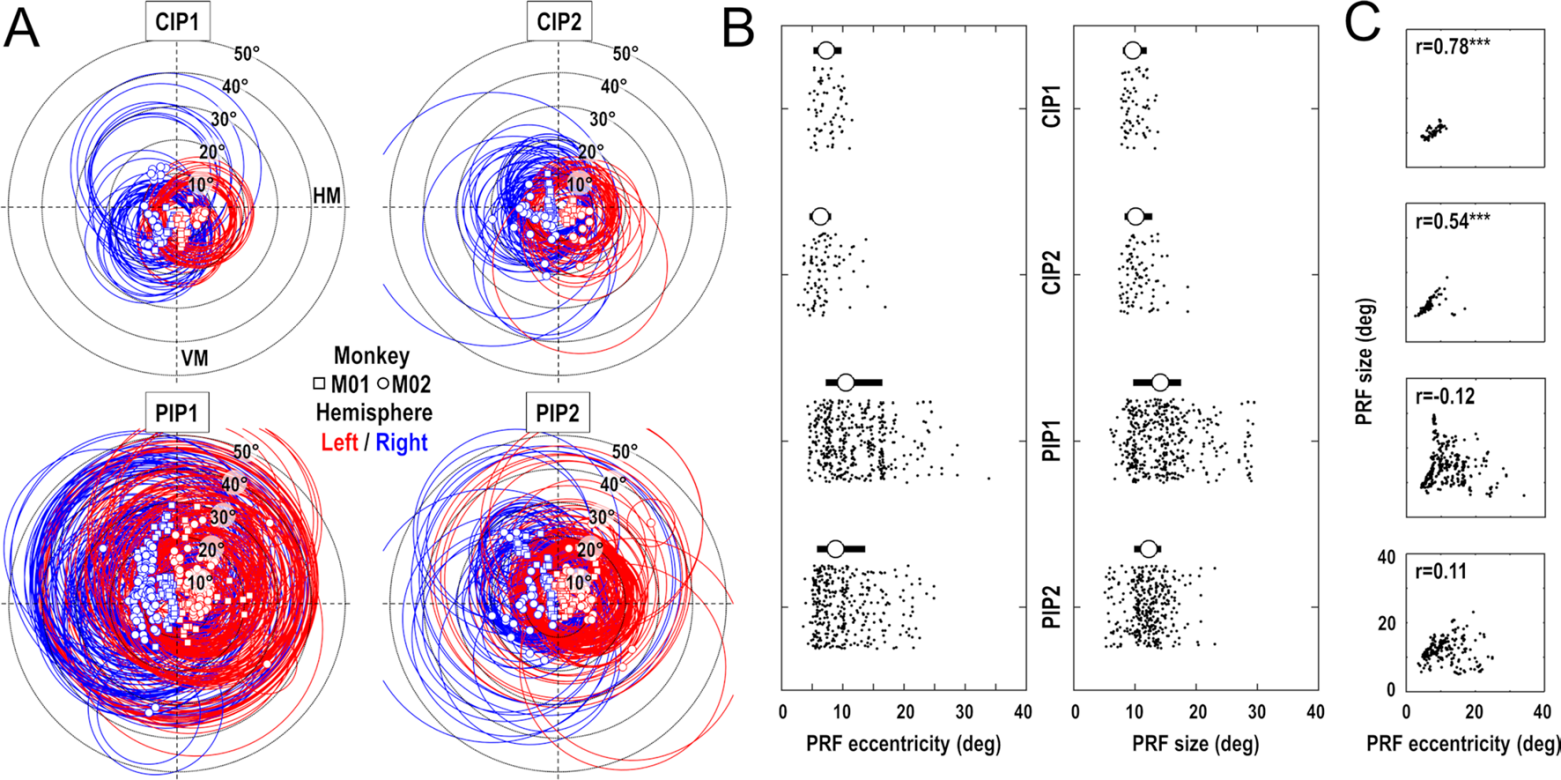
Population receptive fields in the PIP cluster. **a** PRFs distributions in CIP1/CIP2/PIP1/PIP2. The squared and circular symbols indicate the PRF centers of M01 and M02 respectively, while circles show their perimeter (FWHM). The red/blue color code indicate the PRFs from the left and right hemispheres respectively. (HM: horizontal meridian; VM: vertical meridian). **b** Distributions of PRFs eccentricities (left panel) and sizes (right panel) in the 4 areas of the PIP cluster. Circular symbols and thick horizontal lines represent the median sand interquartile ranges. **c** Comparisons of PRFs eccentricities and sizes in the 4 areas, with their correlation of coefficient (r)

We assessed the visual field coverage of the 4 areas constituting the PIP cluster, taking into account both the position and extent of the estimated pRFs. As shown in Fig. 6a, the coverage appears to be more restricted in CIP1 and CIP2 than in PIP1 and PIP2. Two reasons explain this fact. On the one hand, the pRF centers (squared and circular symbols for M01 and M02 respectively) show a smaller range of eccentricity in CIP1 (mean ± 95% CI: 7.5° ± 0.5°) and CIP2 (6.9° ± 0.5°) than in PIP1 (11.8° ± 0.5°) and PIP2 (10.2° ± 0.5°). On the other hand, the pRF sizes (red and blue circles for the left and right hemispheres respectively) are also smaller in CIP1 (10.0° ± 0.4°) and CIP2 (10.7° ± 0.5°) than in PIP1 (14.7° ± 0.5°) and PIP2 (12.1° ± 0.3°). The distributions of pRF eccentricities and sizes are shown in Fig. 6b, with their median and interquartile range. Non-parametric Kruskall–Wallis tests confirm that these differences are highly significant, both for the pRF eccentricities (Chi square = 126.9; *p* < 10^−26^) and for their sizes (Chi square = 122.0; *p* < 10^−25^). We also estimated the visual field coverage of each area by summing all the Gaussian pRFs and calculating the area containing 95% of this density function. Not surprisingly, the horizontal coverage is smaller in CIP1 (− 24.3° to + 21.8°) and in CIP2 (− 27.0° to + 24.1°) than in PIP1 (− 37.2° to + 36.4°) and PIP2 (− 31.4° to + 29.1°). This observation holds for the vertical coverage, but that latter additionally points to a wider coverage of the inferior visual hemifield in CIP1 (− 26.4° to + 19.3°) and CIP2 (− 27.2° to + 22.4°) and to an inverse tendency in PIPI1 (− 32.8° to + 39.9°) and PIP2 (− 25.6° to +32.2°). Overall, we note that these values argue for the need to use wide-field stimuli for studying the visuotopic organization of these parietal areas. Supplementary Fig. 6 illustrates this point by showing that the classical extent of retinotopic stimuli (± 10° to 15°) does not allow a proper mapping of these parietal areas. This point is important as it may explain why Arcaro et al. (2011) could not identify PIP1 and PIP2, the 2 areas with the larger pRF and wider visual field coverage, as well the foveal confluence that links them to CIP1 and CIP2. A final observation that further distinguishes CIP1/2 from PIP1/2 is the correlation between pRFs sizes and eccentricities that is observed only in the former areas (Fig. 6c).

**Fig. 7:**
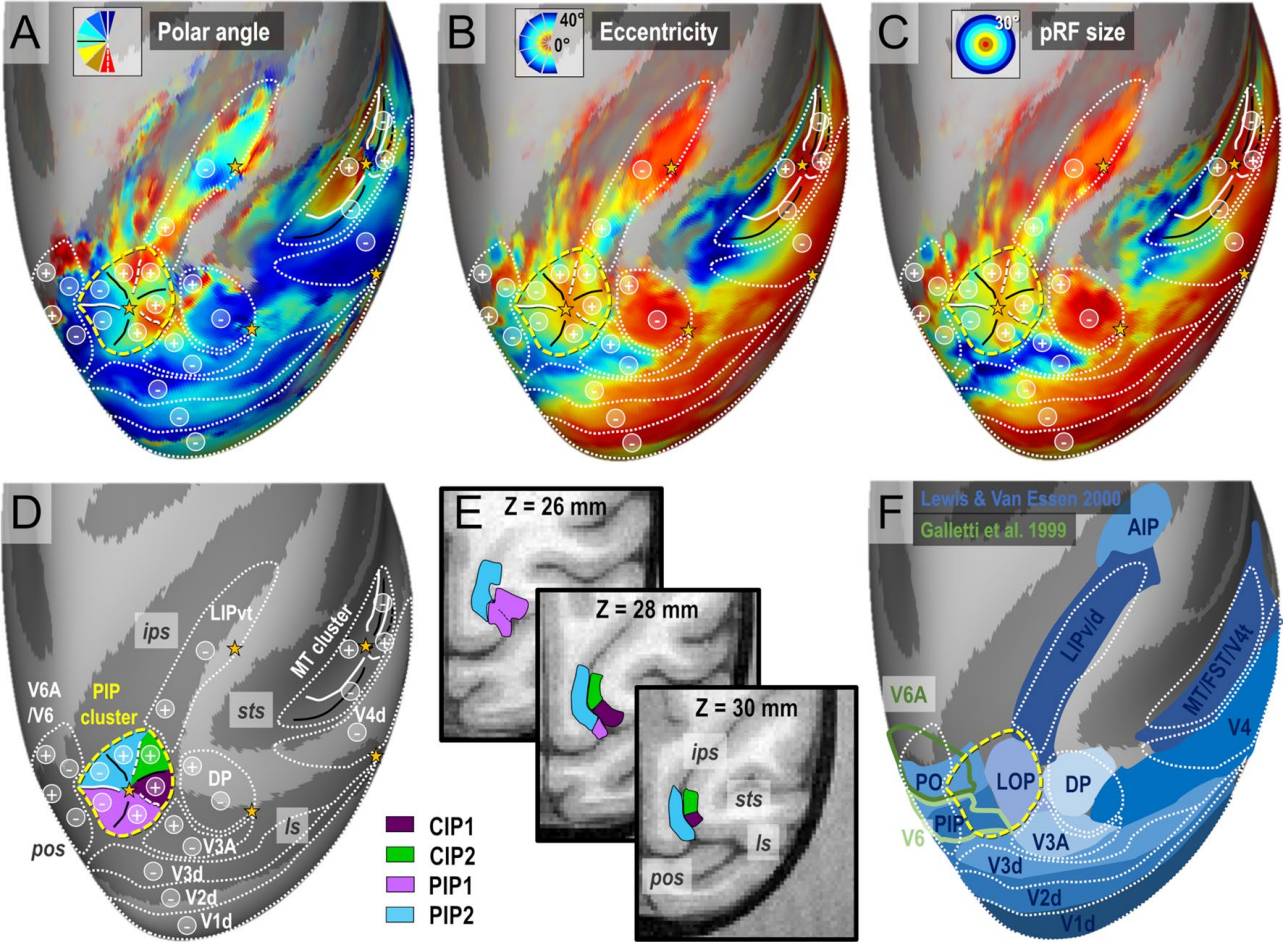
Mean PRF results (4 hemispheres) projected on the right inflated cortical surface of the F99 monkey template. **a** Mean polar angle map. **b** Mean eccentricity map. **c** Mean population receptive fields’ sizes. **d** Outline of the visual areas and visual clusters. **e** Horizontal sections showing the anatomical locations of the PIP cluster’s areas. **f** Comparison of our visuotopic parcellation with the F99 locations of various dorsal visual areas (Lewis et Van Essen 2000) and V6/V6A areas (Galletti et al. 1999a, b). The “+” and “−” bullets indicate representations of the superior and inferior quarter-fields respectively

How does the visuotopic organization of this newly identified cluster integrates with that of the neighbouring areas and with previous findings? Figure 7a–c show the average maps of pRF polar angle, eccentricity and size with the corresponding visuotopic parcellation on top of the right cortical hemisphere of the template monkey F99 (see Methods). Overall, we note a very good agreement between our observations and those of previous electrophysiological and imaging studies regarding the arrangement of upper/lower field representations in V3A/DP, V6/V6A and LIP (Galletti et al. 1999a, b; Hamed et al. 2001; Arcaro et al. 2011; Gamberini et al. 2015; Hadjidimitrakis et al. 2019a, b). At the crossroad of these multiple visuotopic areas, we also note that the PIP cluster offers smooth visuotopic transitions thanks to its “cloverleaf” organization (Fig. 7d). The upper visual field representations of CIP1 and CIP2 (signaled by “+” symbols) are bordered by those of V3A/DP laterally, LIP anteriorly and PIP1/PIP2 medially. Concerning the lower visual field representations of PIP1 and PIP2 (“-” symbols), they face those of V3d posteriorly and V6/V6A medially. Overall, we note a very good agreement between our observations and those of previous electrophysiological and imaging studies regarding the arrangement of upper/lower field representations in V3A/DP, V6/V6A and LIP (Galletti et al. 1999a, b; Hamed et al. 2001; Arcaro et al. 2011; Gamberini et al. 2015; Hadjidimitrakis et al. 2019a, b).

Figure 7E illustrates the anatomical location of the four areas constituting this cluster, at the confluence of the lunate, parieto-occipital and intra-parietal sulci. In the ventral most horizontal section of the F99 monkey’s brain (Z = 26 mm), it can be seen that PIP1 and PIP2 occupy the fundus and medial bank of the parieto-occiptal and intra-parietal sulci. In more dorsal sections (Z = 28 and 30 mm), those areas lie in the medial bank of the IPS, while CIP1 and CIP2 occupy the lateral bank. Overall, we observe a very good agreement between our visuotopic parcellation and that of the dorsal visual areas defined by Lewis and Van Essen on the cortical surfaces of monkey F99 (blue areas in Fig. 7f). In agreement with the observations of Arcaro et al. (2011), CIP1 and CIP2 occupy the location of the lateral occipital parietal (LOP) area. The newly discovered areas PIP1 and PIP2 are within the lateral most sectors of areas PIP and PO in the Lewis and Van Essen’s parcellation scheme. However, Galletti et al. (1999a, b) have introduced a finer parcellation of PIP and PO based on functional and anatomical evidences. Their newly defined areas V6 and V6A (green outlines in Fig. 7f) form a parieto-occipital complex showing only a moderate overlap with our newly defined visuotopic areas PIP1 and PIP2.

## Discussion

By using wide-field retinotopy with fMRI in two behaving macaques, we provide evidence for a new visuotopic cluster in a location previously defined histologically and anatomically as the posterior intra-parietal (PIP) area (Colby et al. 1988; Markov et al. 2014). This PIP cluster includes four visuotopic areas sharing a foveal confluence, an organization echoing that of the recently documented MT cluster (Kolster et al. 2009, 2014) and registering into the general category of “cloverleaf” clusters (Brewer and Barton 2012).

The two smallest and lateral-most areas of this cluster, CIP1 and CIP2, have recently been described with classical retinotopy (Arcaro et al. 2011). In accordance with this previous study, we found that they house mainly upper visual field representations and that they are bordered by other visuotopic areas: V3A/DP latero-posteriorly and LIP anteriorly. One has to be careful though in the interpretation of the border between V3A/DP and area CIP1. While we define area V3A as being anterior to V3d, consistently with a number of studies (Van Essen and Zeki 1978; Arcaro et al. 2011), the precise frontiers of this area remain an active point of debate (Gattass et al. 1988; Zhu and Vanduffel 2019). It is not to be excluded that the differences between these different reports, however, arises from divergence in methodology. What seems to be certain however, is the existence of a full field representation in the lunate sulcus and the annectant gyrus, with a continuous UVF representation into the PIP cluster, but separated from it by an eccentricity/pRF size ridge. Also, we have to note a major difference between our findings and those of Arcaro et al. however, which is the location of the shared CIP1/2 fovea. While they describe it to lie on the lateral bank of sulcus, our results indicate that it lies more medially, in the fundus of the sulcus, and as being the foveal origin of a “cloverleaf” cluster, shared with two other areas, PIP1 & PIP2. These two largest and medial most areas of the cluster are newly discovered visuotopic areas with complete representations of the contralateral hemi-field. They are bordered posteriorly and medially by V3d/V6/V6A, whose visuotopic organizations are consistent with the descriptions drawn from single cell recordings (Gamberini et al. 2015; Hadjidimitrakis et al. 2019a, b). Thus, by introducing wide-field mapping, the present study complements the previous mapping study of Arcaro et al. (2011) and offers a more exhaustive view of the visuotopic maps paving the PPC, notably by documenting a new visuotopic cluster and its relationship with surrounding visuotopic areas. One can point that in humans too, wide-field mapping has been shown to be necessary for detecting the potential human homologues of V6 (Pitzalis et al. 2006) and V6A (Pitzalis et al. 2013).

We have little elements to provide regarding the functional role(s) of CIP1/2 and PIP1/2, except that they do not seem to be particularly involved in processing visual motion (Héjja-Brichard et al. 2020), by contrast with the neighboring V6/V6A complex. Future investigations will have to clarify this issue, but we can nevertheless speculate that CIP1 and/or CIP2 are likely to process static 3D slants defined by binocular disparity or other depth cues (Tsutsui et al. 2005; Durand et al. 2007; Rosenberg et al. 2013). Interestingly, neurons recorded in a location matching that of CIP1/2 have been shown to possess large receptive fields (10°–30°) (Tsutsui et al. 2005), in good agreement with the pRF sizes found in the present study. However, those single cell recordings failed to note any visuotopic organization, illustrating the higher sensitivity of fMRI for revealing large scale organization of the receptive fields in high-order areas (Patel et al. 2010). Recent findings suggest that PIP1 and/or PIP2 might also be involved in visuospatial functions (Premereur et al. 2015; Van Dromme et al. 2016), most notably in cyclopean stereomotion processing (Héjja-Brichard et al. 2020).

Together, our results demonstrate that in macaques, as in humans, the PPC is largely visuotopic. Further engaging in a direct comparison between the large-scale organization of visuotopic maps between human and monkey PPC would remain very speculative, because the only human studies that have employed wide-field retinotopy have focused on the potential human homologues of V6 (Pitzalis et al. 2006) and V6A (Pitzalis et al. 2013). However, we hypothesize, due to corresponding cortical locations and similar functional properties, that area POIPS/CSM_PPC_ (Georgieva et al. 2009; Héjja-Brichard et al. 2020) in humans might correspond to the newly identified PIP1/PIP2 areas. On the other hand, area V7/VIPS/IPS0 (Press et al. 2001; Georgieva et al. 2009; Konen et al. 2013) might be the human homolog of areas CIP1/CIP2, of which the retinotopic and function characteristics (Tsao et al. 2003; Shikata et al. 2008) are strongly reminiscent of those of monkey CIP.

While we did not detect a clear demarcation between V3d and V6, we did detect robust motion sensitivity in the piece of parieto-occipital cortex in which V6/V6A has been previously reported. Additionally, our data reveals that V6/V6A possess representations of far eccentricities (> 20°), that they share borders with V3d and the PIP cluster, and that they are separated by an eccentricity gradient inversion. Non-generalizable, yet non negligible evidence allows us to speculate on a possible demarcation between V6 and V3d: in most hemispheres, the horizontal meridian that constitutes the limit between V2d and V3d is cut by a representation of the UVM. This occurring severance also seems to overlap with the limit of V6′s motion sensitivity. Although no safe conclusions can be drawn from this observation, and considering a recent report (Hadjidimitrakis et al. 2019a, b) suggesting a continuous V3d/V6 eccentricity gradient, our findings encourage further investigation. It is interesting to indicate that for most of the visuotopic maps already documented in human PPC, the delineations rely almost exclusively on polar angle gradient inversions, since foveal representations and eccentricity gradients are notoriously ambiguous (Brewer and Barton 2012). Our study provides a good illustration that beyond the potential to divulge novel visuotopic maps, wide-field retinotopy also disambiguates the location of foveal representations and the direction of eccentricity gradients.

To conclude, our results deliver the most exhaustive view to date of the visuotopic organization of monkey PPC. They show a dense organization of abutting visual field maps, of which some are arranged in clusters. Generalizing wide-field stimulation during functional investigation of visual areas in both species, will undoubtedly contribute to the thorough understanding of these large-scale visuotopic ensembles. This represents not only a primordial goal for understanding the relationship between visuotopic representation and function, but also an essential step in the quest for establishing the functional homologies between the PPC of human and non-human primates (Orban et al. 2004, 2006; Sereno and Tootell 2005).

## Electronic supplementary material

Below is the link to the electronic supplementary material.

Supplementary material 1 (DOCX 10116 kb)
