# Supplementary material for: Wide-field retinotopy reveals a new visuotopic cluster in macaque posterior parietal cortex
Source: Brain Struct Funct. 2020 Sep 2;225(8):2447–61. doi: 10.1007/s00429-020-02134-2 (PMC7544618; doi:10.1007/s00429-020-02134-2)
Supplement: Supplementary file 1 — Supplementary material 1 (DOCX 10116 kb) [file 429_2020_2134_MOESM1_ESM.docx]

**SUPPLEMENTARY MATERIAL**

**Widefield retinotopy reveals
a new visuotopic cluster
in macaque posterior parietal cortex**

Samy Rima^1,2*^, Benoit R. Cottereau^1,2^, Yseut Héjja-Brichard^1,2^, Yves Trotter^1,2^ & Jean-Baptiste Durand^1,2*`^

^1^ Université de Toulouse, Centre de Recherche Cerveau et Cognition, Toulouse, France

^2^ Centre National de la Recherche Scientifique, Toulouse Cedex, France

* Corresponding author (samy.rima@unifr.ch; jbdurand@cnrs.fr)

**Supplementary Figure 1: Illustration of retinotopic data processing**

**Supplementary Figure 2: Maps of PRFs goodness of fit**

**Supplementary Figure 3: Test-retest analysis of the PRF model parameters**

**Supplementary Figure 4: Individual maps of PRF sizes**

**Supplementary Figure 5. Eccentricity-dependent polar path analysis**

**Supplementary Figure 6. PRF size/eccentricity versus stimuli coverage**

**
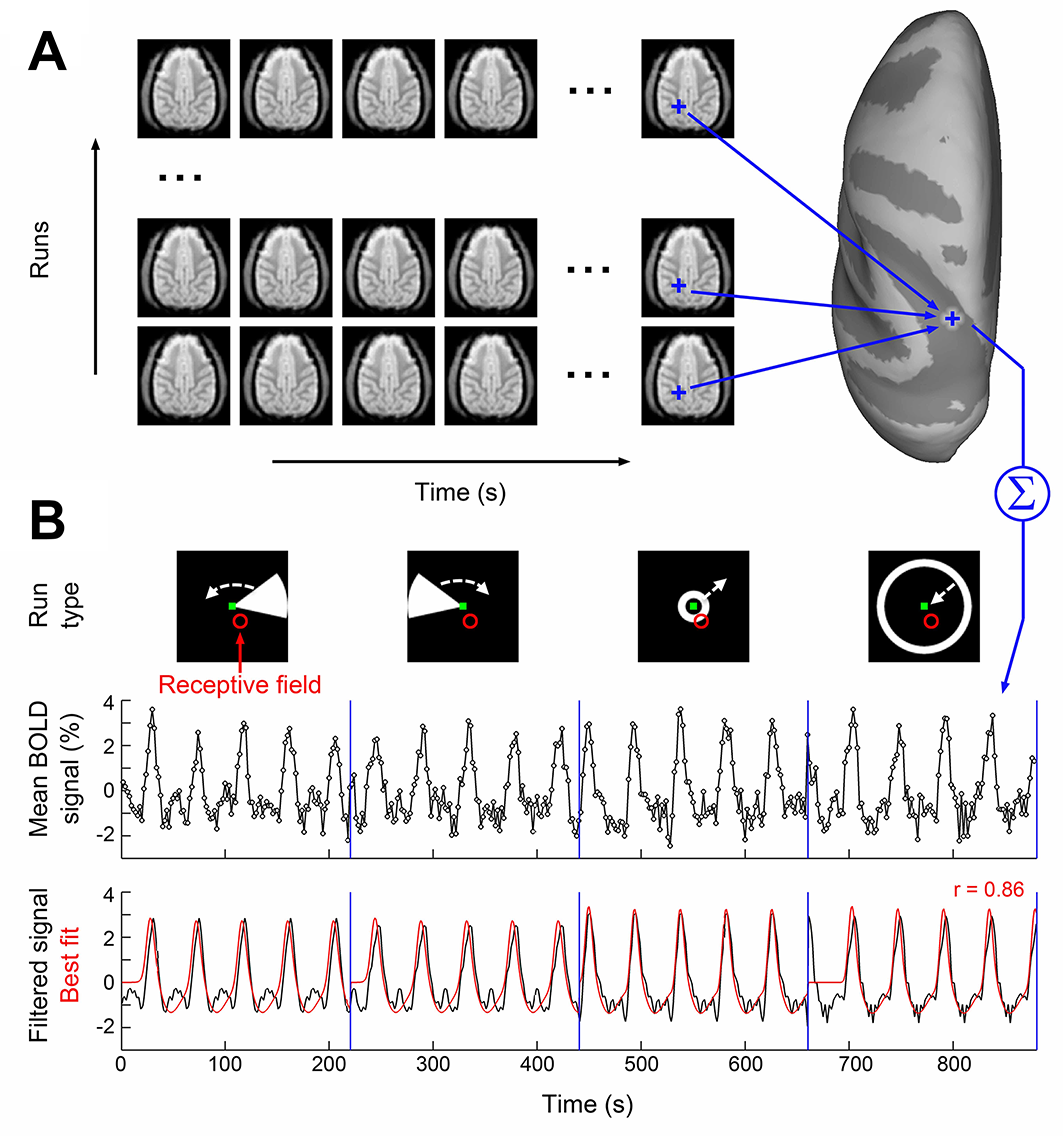
**

**Supplementary Figure 1. Illustration of retinotopic data processing.** **(A)** After pre-processing, functional volumes were projected onto the reconstructed cortical surfaces and averaged to produce mean BOLD time courses, after conversion to percent signal change unit (see Material and Methods). **(B)** For the 4 types of run shown in the upper panels (clockwise and counter-clockwise wedges, expanding and contracting rings), the mean BOLD time courses (black curve in the middle panel) were Fourier filtered to retain only the signal components corresponding to the fundamental frequency of the periodic stimuli and all its harmonics (black curve in the lower panel). The PRF analysis resulted in selecting the model time course (red curve in the lower panel) exhibiting the highest correlation with this filtered signal. If the correlation coefficient was superior to 0.5 (r=0.86 in the present example), the PRF parameters attached to the theoretical time course (polar angle, eccentricity and size) were retained. In the present example, those parameters define the PRF shown in red in the upper panels.

**
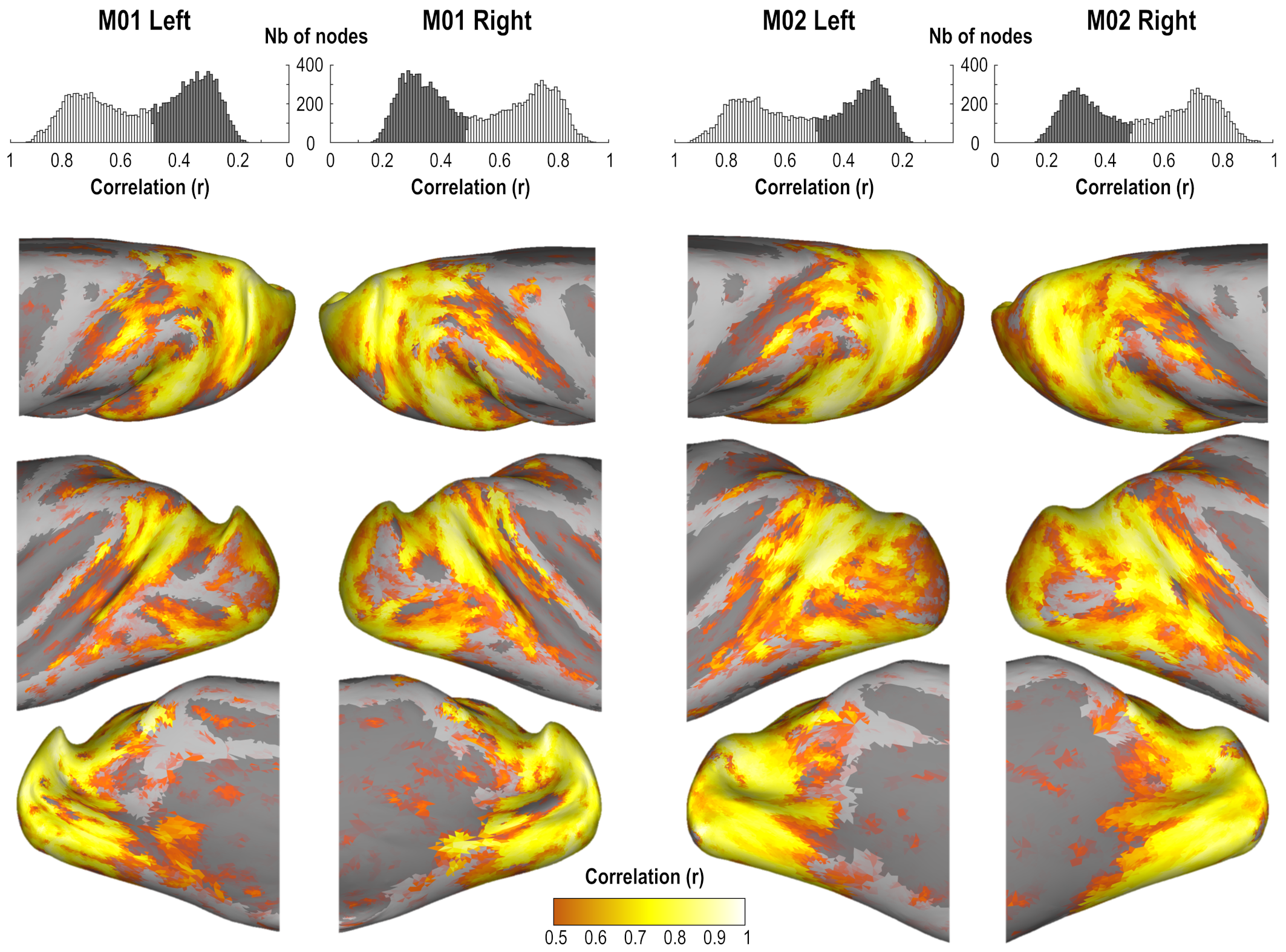
**

**Supplementary Figure 2. Maps of PRFs goodness of fit.** The top row shows the distribution correlation coefficients (r) across nodes for each of the 4 reconstructed cortical surfaces. Only nodes for which r>0.5 (corresponding to an uncorrected p value < 10^-6^) were retained for further analysis. The distribution of those nodes is shown on dorsal, lateral and medial views of the 4 reconstructed cortical surfaces, color coded as a function of their r value.

**
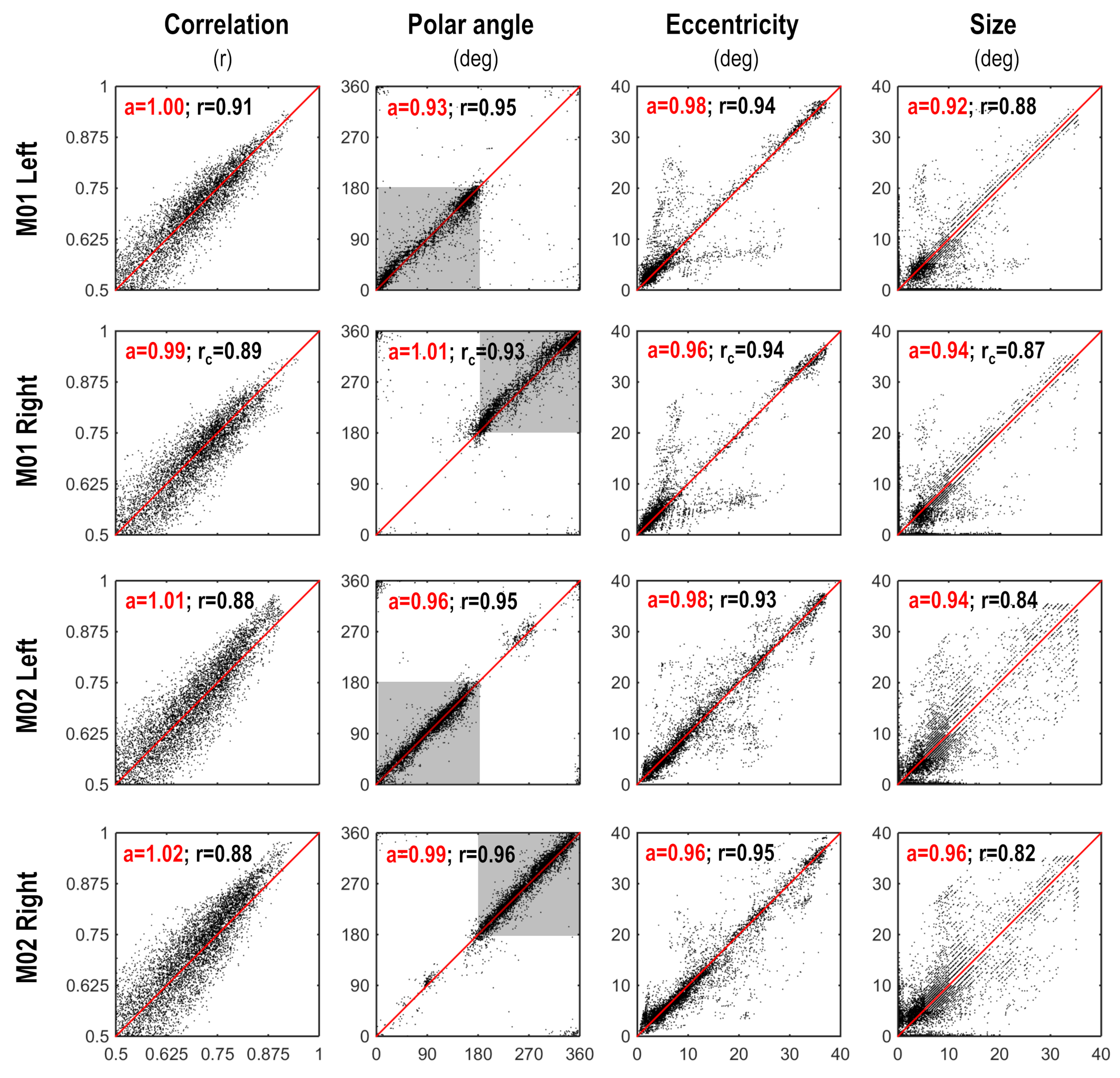
**

**Supplementary Figure 3. Test-retest analysis of the PRF model parameters.** The PRF analysis was performed separately on the odd and even runs and the model parameters obtained with these two independent data set were compared. Comparison of the correlation coefficient (r), polar angles, eccentricities and receptive field sizes show a great level of reproducibility in all 4 cortical hemispheres, as indicated by correlation values and slopes of the regression line slopes which are all close to unity. For the polar angle values, circular correlation (r_c_) was used and the grey zones indicate contro-lateral space (i.e., left visual hemi-field for the right cortical hemispheres and right hemi-field for left hemispheres). The percentages of nodes in contralateral space or very close to it (delta polar angle < 5° or eccentricity <2°) were 97.8% and 99.0% for the left and right hemispheres of M01 (LH M01 and RH M01), and 96.4% and 97.4% for the left and right hemispheres of M02 (LH M02 and RH M02).


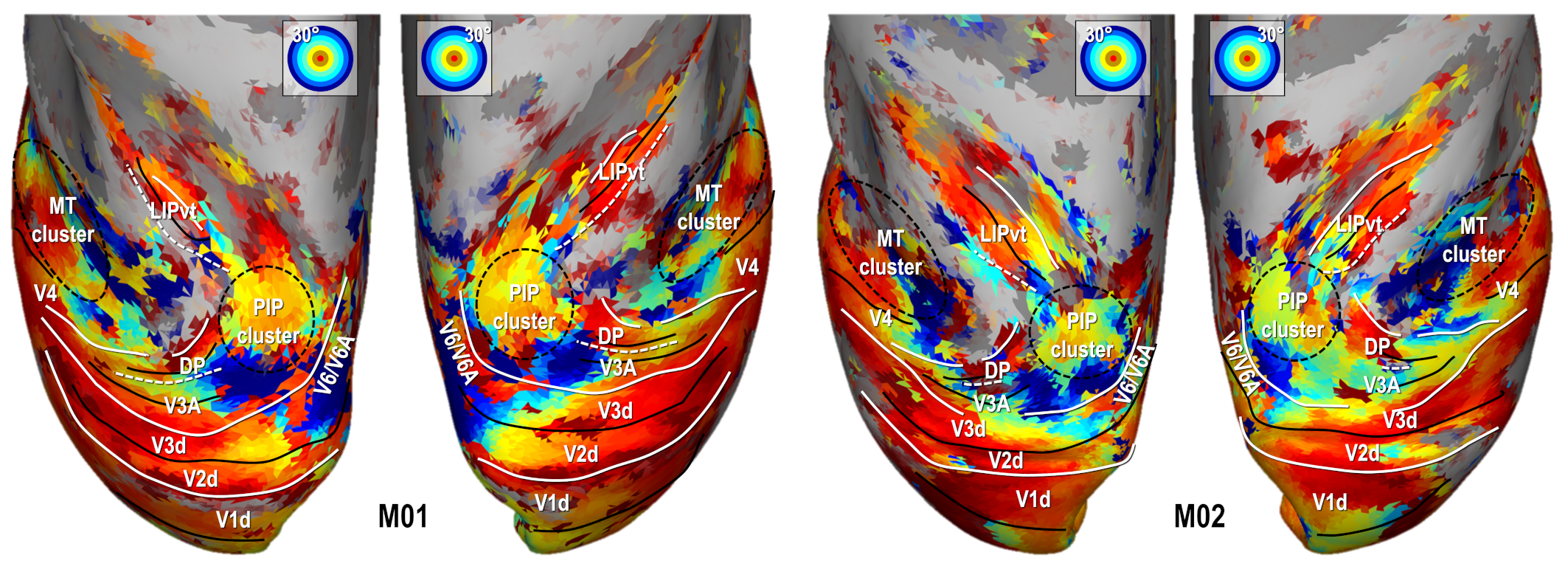


**Supplementary Figure** 4. **Individual maps of PRF sizes.** The color code on top of the individual cortical surface reconstructions for M01 and M02 indicates the estimated pRF sizes (threshold: r>0.5), from 0.25° in red to 30° in blue.

**
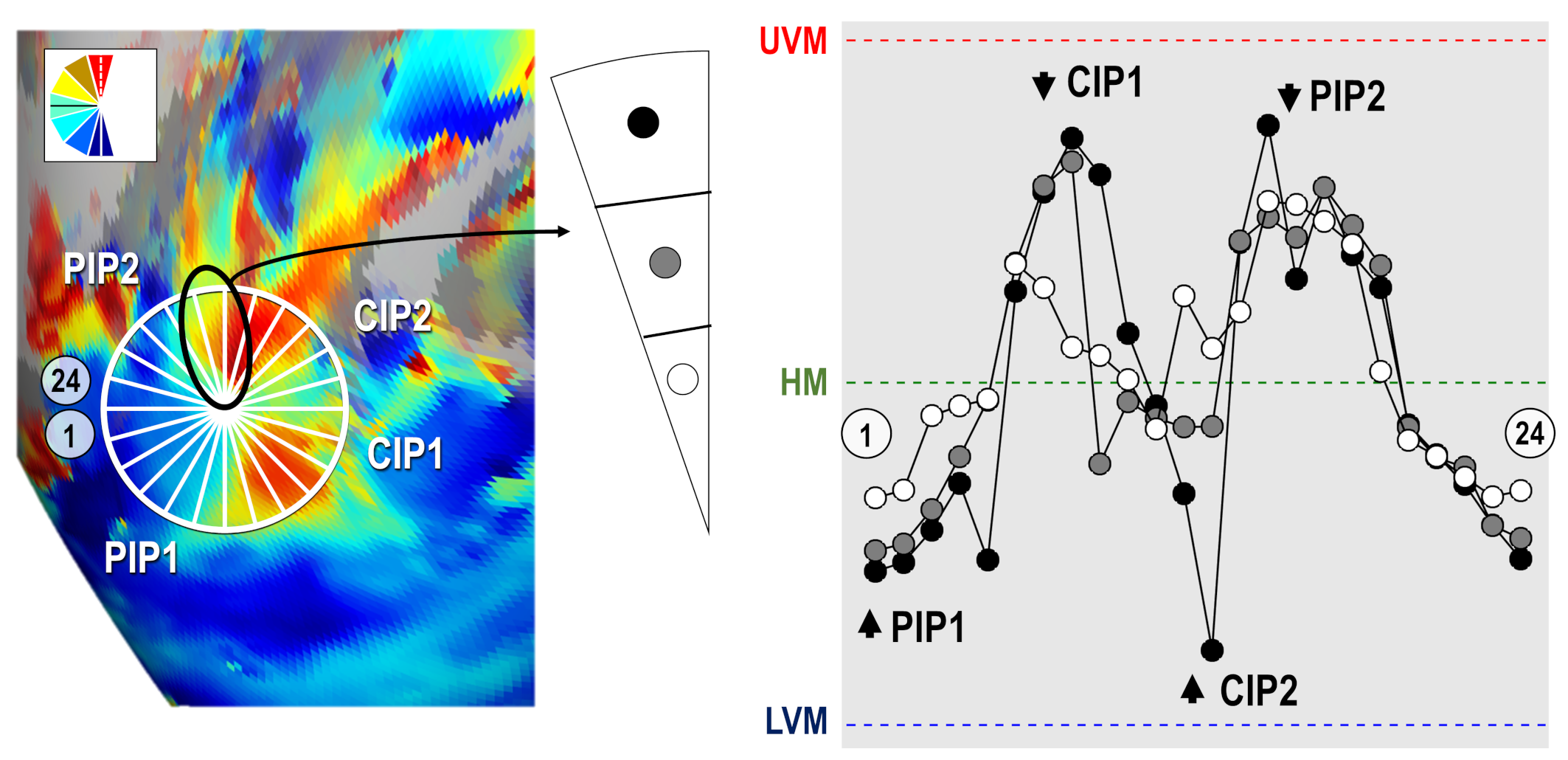
**

**Supplementary Figure 5. Eccentricity-dependent polar path analysis.** Same conventions as Figure 5B, except that 3 circular polar angle profiles were drawn by differentiating the nodes in 3 equal sectors according to their close, intermediate or long distance from the shared foveal representation.

**
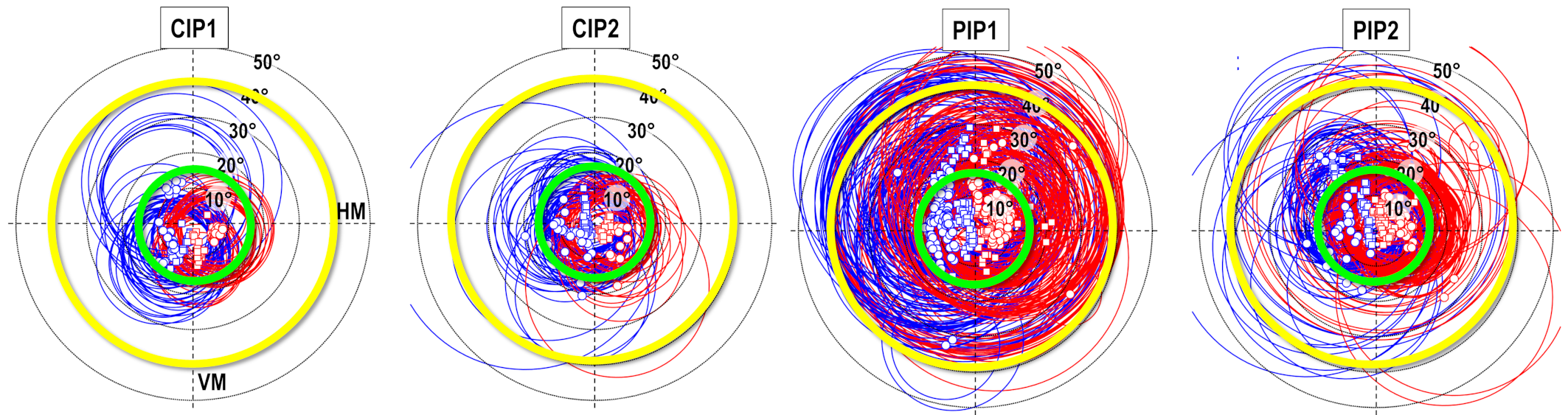
**

**Supplementary Figure 6. PRF size/eccentricity versus stimuli coverage.** PRFs of the 4 areas of the PIP cluster, as shown in Figure 6. The yellow circle in the foreground indicates the size of the retinotopic stimuli used in the present study (80°). The green circle shows the size of the stimuli (30°) used by Arcaro and collaborators (2011).
